# Differential diagnosis of sinonasal extranodal NK/T cell lymphoma and diffuse large B cell lymphoma on MRI

**DOI:** 10.1007/s00234-020-02471-3

**Published:** 2020-06-19

**Authors:** Yun Chen, Xinyan Wang, Long Li, Wei Li, Junfang Xian

**Affiliations:** 1 Department of Radiology, Beijing Tongren Hospital, Capital Medical University, Beijing, 100730 China; 2 Department of Radiology, Third Affiliated Hospital of Soochow University, Changzhou, Jiangsu China; 3 Department of Radiology, Xianghe People’s Hospital, Langfang, Hebei China

**Keywords:** Magnetic resonance imaging, Lymphoma, Paranasal sinus, Nasal cavity

## Abstract

**Purpose:**

To evaluate whether imaging features on conventional magnetic resonance imaging (MRI) can differentiate sinonasal extranodal natural killer/T cell lymphomas (ENKTL) from diffuse large B cell lymphoma (DLBCL).

**Methods:**

Consecutively, pathology-proven 59 patients with ENKTL and 27 patients with DLBCL in the sinonasal region were included in this study. Imaging features included tumor side, location, margin, pre-contrast T1 and T2 signal intensity and homogeneity, post-contrast enhancement degree and homogeneity, septal enhancement pattern, internal necrosis, mass effect, and adjacent involvements. These imaging features for each ENKTL or DLBCL on total 86 MRI scans were indicated independently by two experienced head and neck radiologists. The MRI-based performance in differential diagnosis of the two types of lymphomas was evaluated by multivariate logistic regression analysis.

**Results:**

All ENKTLs were located in the nasal cavity, with ill-defined margin, heterogeneous signal intensity, internal necrosis, marked enhancement of solid component on MRI, whereas DLBCLs were more often located in the paranasal sinuses, with MR homogenous intensity, mild enhancement, septal enhancement pattern, and intracranial or orbital involvements (all *P* < 0.05). Using a combination of location, internal necrosis and septal enhancement pattern of the tumor in multivariate logistic regression analysis, sensitivity, specificity, and accuracy in differential diagnosis of ENKTL and DLBCL were 100%, 79.4%, and 91.9%, respectively, for radiologist 1, and were 98.3%, 81.5%, and 93.0%, respectively, for radiologist 2.

**Conclusion:**

MRI can effectively differentiate ENKTL from DLBCL in the sinonasal region with a high diagnostic accuracy.

## Introduction

**N**on-Hodgkin lymphoma (NHL) is the second most common primary malignancy in the sinonasal region, accounting for about 12–17% of all sinonasal cancers [1]. The most two common subtypes of NHL associated with the sinonasal region are extranodal natural killer/T cell lymphoma (ENKTL) and diffuse large B cell lymphoma (DLBCL) [1, 2]. In geographical distribution, ENKTL of nasal type is the most common type of lymphoma in Asia and Latin America, whereas DLBCL is more common in Western countries [1, 2]. Originally, ENKTL arises predominantly from natural killer cell and DLBCL arises from mature B lymphocytes. Treatment strategies for the two histologic subtypes of lymphomas are different [3–5]. For sinonasal DLBCL, the chemotherapy treatment known as R-CHOP (rituximab-cyclophosphamide, doxorubicin, vincristine, and predisone) is most common [3, 4]. On the other hand, the current standard approach for sinonasal ENKTL is the non-anthracycline-containing chemotherapy with or without radiotherapy [5]. Moreover, prognosis for ENKTL is worse than that for DLBCL due to its aggressive course with marked destructive capacity [6, 7]. Case-controlled disease-specific survival analysis showed that the 5-year disease-specific survival (DSS) for patients with sinonasal DLBCLs was 72.8%, compared with only 38.4% for patients with ENKTLs [6]. Therefore, it is very important to differentiate sinonasal ENKTL from DLBCL for managing appropriate treatment plans to improve patient survival.

The differential diagnosis of sinonasal ENKTL and DLBCL can be highly challenging if it is only based on clinical signs and symptoms. The clinical presentation of patients with sinonasal ENKTL or DLBCL is nonspecific, mimicking allergic rhinitis and upper respiratory infection [7]. Furthermore, the diagnostic performance of biopsy for sinonasal lymphoma is poor due to an uncertainty whether correct tissue is sampled [8]. One main reason is because that sinonasal ENKTL formerly known as lethal midline granuloma may present with a necrotizing and angiodestructive growth pattern, and an extensive coagulative necrosis in the tumor can be misdiagnosed as inflammatory process [8]. According to a previous study, the sensitivity of endoscopic incisional biopsy in differential diagnosis of necrotizing sinonasal lesions is extremely low [9].

Magnetic resonance imaging (MRI) has been proven to play crucial roles in the differential diagnosis of sinonasal tumors [10, 11], especially for classification of the lymphoma and squamous cell carcinoma [12–14]. Previous studies reported that the homogenous signal intensity, facial soft tissue involvement, and low ADC values on MR findings suggested the diagnosis of the lymphomas [12–14]. However, whether these features can help in differential diagnosis of lymphoma subtypes is still unclear. To our knowledge, only a few studies discussed the usefulness of DWI values combined with other MRI parameters in differential diagnosis of ENKTL and DLBCL [15].

The purpose of this current study was to evaluate whether conventional MR imaging features can differentiate sinonasal ENKTL from DLBCL.

## Materials and methods

Study approval was granted by the institutional review board and the informed consent was waived for this retrospective study.

### Patients

This retrospective study included consecutive MRI scans of 59 patients (41 men and 18 women; mean age 46 ± 15 years) with sinonasal ENKTL and 27 patients (16 men and 11 women; mean age 66 ± 13 years) with sinonasal DLBCL examined during February 2011 and November 2018. Inclusion criteria: (1) sinonasal mass demonstrated at MRI proved to be ENKTL or DLBCL by pathologic examination and (2) the MRI scans were performed prior to the biopsy or any treatments such as radiotherapy or chemotherapy. MRI scans with either a recurrent ENKTL or DLBCL were excluded from this study.

### MR protocol

Conventional MRI scans were performed in 36 patients with GE HDxt 3.0-T MR scanner (GE Healthcare, Milwaukee, WI) and 50 patients with 3.0-T Discovery 750 scanner (GE Healthcare, Milwaukee, Wisconsin, USA) by using an 8-channel head coil. Pre-contrast axial T1WI and T2WI as well as post-contrast fast spin echo (FSE) T1WI on axial, coronal, and sagittal view were performed in all patients. The parameters were as follows: FSE T1WI (TR 600~700 ms,TE 10 ms, matrix 320 × 256), FSE T2WI (TR 3900~4300 ms, TE 90 ms, matrix 512 × 256), FOV 20 × 20 cm, slice thickness 4~5 mm, gap 1 mm. Gadopentetate dimeglumine contrast agent (Magnevist; Bayer Schering, Berlin,Germany) was administered intravenously (0.1 mmol/kg) at a flow rate of 2 mL/s, followed by a 20-mL saline flush.

### Imaging analysis

**Fig. 1:**
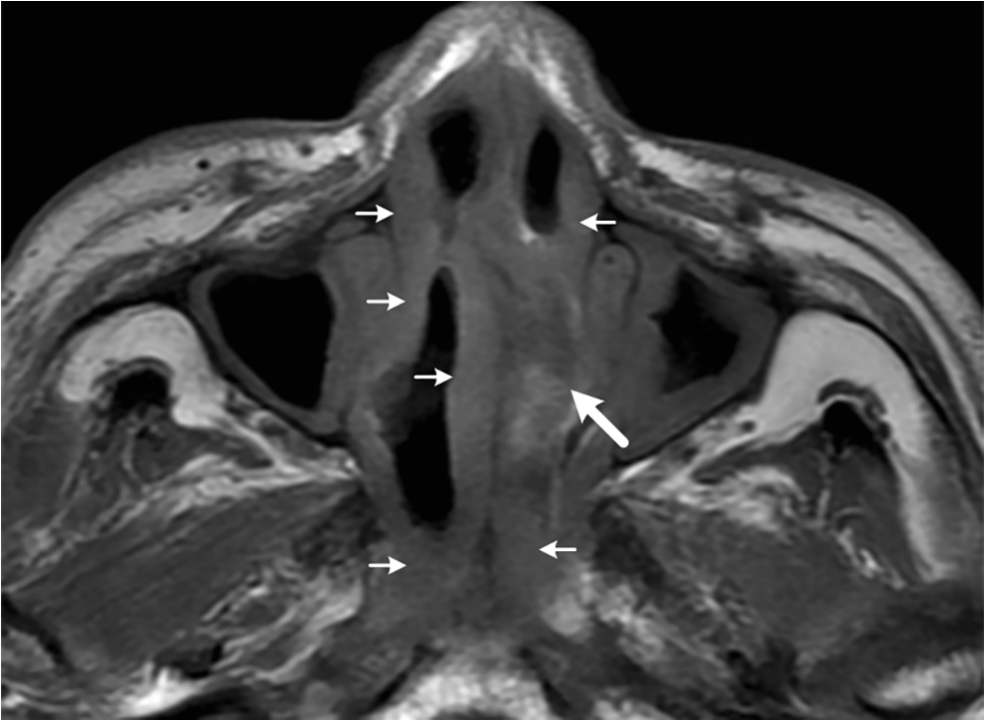
An example for the septal enhancement pattern. Axial delayed contrast T1WI shows marked enhanced striations (arrows) within mild enhanced solid components of the tumor

Imaging features per each sinonasal ENKTL or DLBCL on 86 MRI scans were indicated by two experienced radiologists independently (**X.Y.W. and Y.C**, with 10 and 16 years of experience in head and neck imaging, respectively), without the information of final pathology diagnosis. The MRI imaging features of the tumors included side, location, margin, pre-contrast T1 and T2 signal intensity and homogeneity, post-contrast enhancement degree and homogeneity, septal enhancement pattern, internal necrosis, mass effect, and adjacent involvements. Three levels of the enhancement degree were defined as (1) mild (similar to adjacent muscles except extraocular muscles), (2) moderate (greater than muscles), and (3) marked (similar to or greater than nasal mucosa). Further, a septal enhancement pattern was considered if partially or diffusely marked enhanced striations could be seen within lower- or non-enhanced components of the tumor on delayed contrast T1WI (Fig. 1).

### Statistical analysis

All statistical analyses were performed with the IBM SPSS 20.0 software. Differences in MRI features between ENKTL and DLBCL were determined by Chi-square test or Fisher’s exact test. The *P* value less than 0.05 was considered significant. Inter-reader reproducibility of all evaluated MRI features was evaluated by kappa analysis. The kappa values were interpreted as < 0.40, poor; 0.41–0.60, moderate; 0.61–0.80, good; and > 0.81, excellent. Multivariate logistic regression analysis was applied to evaluate MRI-based performance in the differential diagnosis of sinonasal ENKTL and DLBCL.

## Results

**Fig. 2:**
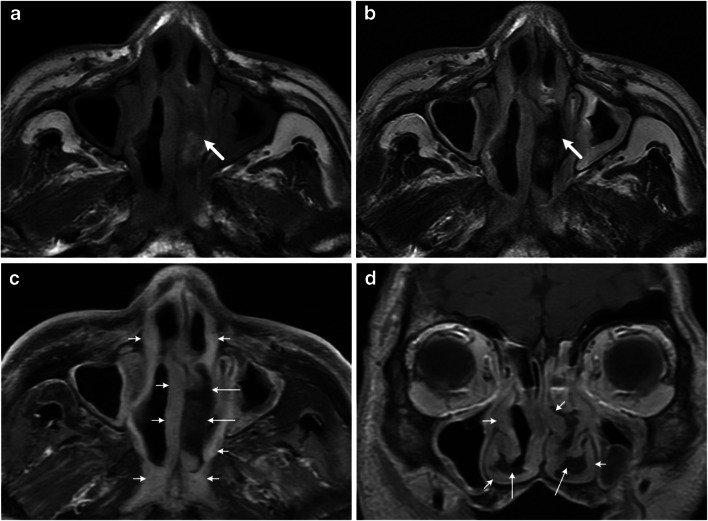
MRI features in a 67-year-old man with pathologically proven extranodal natural killer/T cell lymphoma (ENKTL) in the sinonasal region. a Axial T1-weighted MR image shows diffuse subcutaneous soft tissue thickening (small short arrows) in the bilateral nasal cavities and nasopharynx and an elongated mass (arrow) in the left nasal cavity with heterogeneous signal intensity. **b** Axial T2-weighted MR image shows a heterogeneous isointense tumor (arrow) with hypointense foci. **c**, **d** Axial (**c**) and coronal (**d**) contrast-enhanced T1-weighted MR images show the thickening subcutaneous soft tissue (small short arrows) with marked enhancement; no enhancement part was considered as the internal necrosis (small long arrows)

**Fig. 3:**
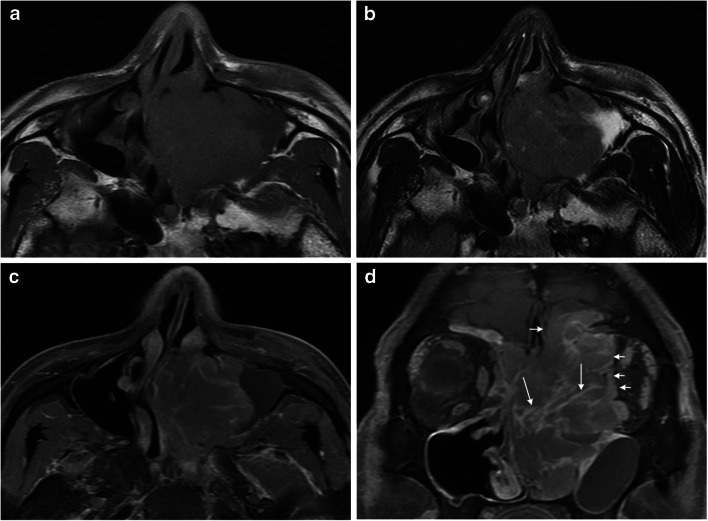
MRI features in a 46-year-old man with diffuse large B cell lymphoma in the sinonasal region. **a** Axial T1-weighted MR image shows a homogeneously isointense mass in left nasal cavity and maxillary sinus. **b** Axial T2-weighted MR image shows the tumor with homogeneous isointensity. **c**, **d** Axial (c** a**) and coronal (**d**) contrast-enhanced T1-weighted MR images show heterogeneous enhancement of the tumor with the septal enhancement pattern (small long arrows) and the involvement of the skull and left orbit (small short arrows)

MR imaging features of sinonasal ENKTL and DLBCL and interobserver agreement between observers 1 and 2 are shown in Table 1. Significant differences were found in location, margin, T1 and T2 homogeneity, degree of enhancement, enhancement homogeneity, septal enhancement pattern, mass effect, internal necrosis, and adjacent structure involvements (all *P* < 0.03). All the ENKTLs were located in the nasal cavity, whereas DLBCLs were more often located in the paranasal sinuses (67%). Furthermore, ill-defined margin, marked enhancement, heterogeneous signal intensity, internal necrosis, less mass effect, and ala nasi involvement were often seen in sinonasal ENKTL (Fig. 2), whereas homogenous signal intensity, mild enhancement, septal enhancement pattern, and intracranial and orbital involvements were often seen in sinonasal DLBCL (Fig. 3).

**Table 1:** MRI features of sinonasal extranodal NK/T cell lymphomas and diffuse large B cell lymphomas

		ENKTL (*n* = 59)		DLBCL (*n* = 27)			
		No.	%	No.	%		
No. of patients		59	100	27	100		
Side						0.450	1
	Left	17	29	8	30		
	Right	17	29	11	41		
	Bilateral	25	42	8	30		
Location						*0.000*	0.964
	Nasal cavity	59	100	9	33		
	Paranasal sinus	0	0	18	67		
Margin						*0.000*	0.757
	Well defined	6	10	19	70		
	Ill defined	53	90	8	30		
T1 signal intensity						0.702	0.845
	Hypointense	5	8	3	11		
	Isointense	54	92	24	89		
T1 homogeneity						*0.027*	0.54
	Homogenous	39	66	24	89		
	Heterogeneous	20	34	3	11		
T2 signal intensity						0.867	0.691
	Hypointense	5	8	2	7		
	Isodense	54	92	25	93		
	Hyperintense	0	0	2	7		
T2 homogeneity						*0.003*	0.624
	Homogenous	28	47	22	81		
	Heterogeneous	31	53	5	19		
Degree of enhancement						*0.000*	0.785
	Mild	7	12	10	37		
	Moderate	32	54	17	63		
	Marked	20	34	0	0		
Enhancement homogeneity						*0.000*	0.696
	Homogenous	10	17	20	74		
	Heterogeneous	49	83	7	26		
Septal enhancement pattern						*0.000*	0.729
	Yes	0	0	11	41		
	No	59	100	16	59		
Internal necrosis						*0.000*	0.803
	Yes	50	85	4	15		
	No	9	15	23	85		
Mass effect						*0.000*	0.720
	Yes	16	27	22	81		
	No	43	73	5	19		
Facial soft tissue involvement						0.433	0.678
	Yes	21	36	12	44		
	No	38	64	15	56		
Intracranial involvement						*0.000*	0.849
	Yes	2	3	9	33		
	No	57	97	18	67		
Orbital involvement						*0.000*	0.854
	Yes	6	10	18	67		
	No	53	90	9	33		
Nasopharynx involvement						0.310	0.673
	Yes	17	29	5	19		
	No	42	71	22	81		
Ala nasi involvement						*0.000*	0.878
	Yes	44	75	8	30		
	No	15	25	9	33		

*ENKTL* extranodal NK/T cell lymphomas, *DLBCL* diffuse large B cell lymphoma

Using a combination of location, internal necrosis, and septal enhancement pattern in multivariate logistic regression analysis, sensitivity, specificity, and accuracy in differential diagnosis of ENKTL and DLBCL were 100%, 79.4%, and 91.9%, respectively, for radiologist 1, and were 98.3%, 81.5%, and 93.0%, respectively, for radiologist 2.

## Discussion

Some previous studies reported that due to nonspecific physical and radiological features, a definitive diagnosis of NEKTL was challenged in clinical practice, and it usually required repeat and deep biopsy [16]. In the current study, we analyzed MRI features for consecutive 59 patients with ENKTL compared with 27 patients with DLBCL. We found that MRI features showed a reliable and high diagnostic accuracy in differential diagnosis of sinonasal ENKTL and DLBCL.

The sinonasal ENKTL is generally a highly aggressive tumor characterized by vascular damage and destruction [7]. The prognosis of ENKTL is very poor, with 5-year disease-specific survival of about 31~46% [6, 17]. On the other hand, DLBCL has a better prognosis regardless of gender, stage, and age. Based on different prognoses and treatment strategies, we tried to differentiate ENKTL from DLBCL by MRI in this study.

The most useful three features for differential diagnosis of sinonasal ENKTL and DLBCL were the location, internal necrosis, and septal enhancement pattern from our study. Some previous studies reported that most sinonasal ENKTLs were located in the nasal cavity [15, 18]. Consistent to previous studies, all of the 59 ENKTLs in our study were located in the nasal cavity. However, if a tumor is located in the paranasal sinus, the possible diagnosis may be a DLBCL rather than an ENKT. Internal necrosis was more commonly found in ENKTL (85%) than that in DLBCL (15%). Pathological reasons include that the ENKTL has the angiocentric and angiodestructive growth pattern that can easily cause the coagulative necrosis and ulcer, whereas the DLBCL has been characterized by sheets of large cleaved and/or noncleaved cells [19]. Septal enhancement pattern was found in 41% of DLBCLs, while none of ENKTLs. The similar MRI enhancement pattern has been previously reported on malignant lymphomas of the ovary and breast [20, 21]. We believe that this septal enhancement pattern could be not specific for sinonasal DLBCL only, but also might be for some other small round cell tumors that were attributed to fibrous bands.

A previous study by He et al. reported that the significant differences were found in degree of MRI enhancement between sinonasal ENKTL and DLBCL [15]. However, we found that the enhanced degree showed a significant higher prevalence of “marked enhancement” in ENKTLs compared with DLBCLs, whereas the previous study reported that the enhancement was less intense for their ENKTLs. The reasons for this are probably because of different evaluation methods or some bias in subjective observations. We only evaluated the solid part of tumors for the enhancement degree levels (no evaluation for possible necrosis part) in this study.

Our study showed that sinonasal ENKTL tended to have less mass effect than DLBCL (27% versus 81%), which is similar to primary lymphomas in central nervous system [22]. The reason should be because the ENKTL usually diffusely infiltrate along the walls of the nasal cavity, enveloping the nasal turbinate and nasal septum with no obvious mass effect. As same as the adjacent involvement reported by a previous study [15], the prevalence of intracranial or orbit involvements was lower in sinonasal ENKTL than that in DLBCL in our study. Sinonasal ENKTLs located in the nasal cavity with earlier nasal symptoms were more often diagnosed at an early stage, whereas DLBCLs hidden in the paranasal sinuses were more often diagnosed at a late stage [23]. In addition, DLBCL located in the paranasal sinuses was much nearer to the skull and preferred to show intracranial involvement.

Our study had several limitations. First, in our consecutive series, the patients with pathology-proven primary sinonasal DLBCL were relatively a small patient set compared to contemporaneous patients with the ENKTL, and it might cause that only three MRI features could be used in the multiple logistic regression analysis. Second, although bone destruction could be an imaging feature of sinonasal ENKTL suggested by other studies, this feature was not evaluated in this study because we were considering the limited value of MRI in evaluation of bone changes. Finally, the MRI examinations were acquired using two different MRI systems.

## Conclusion

MRI can effectively differentiate ENKTL from DLBCL in the sinonasal region with a high diagnostic accuracy. Sinonasal ENKTL should be considered for differential diagnosis if an ill-defined tumor in the nasal cavity shows moderate-significant enhancement with internal necrosis, less mass effect, or ala nasi involvement.

## Data Availability

The processed data required to reproduce these findings cannot be shared at this time as the data also forms part of an ongoing study.
